# Using judgement analysis to identify dietitians’ referral prioritisation for assessment in adult acute services

**DOI:** 10.1038/ejcn.2017.123

**Published:** 2017-08-23

**Authors:** M Hickson, M Davies, H Gokalp, P Harries

**Affiliations:** 1 Institute of Health and Community, University of Plymouth, Plymouth, UK; 2 Department of Clinical Sciences, Brunel University London, London, UK

## Abstract

**Background/Objectives:**

Dietitians need to prioritise referrals in order to manage their work load. Novice dietitians may not receive training on prioritisation and could be helped with an evidence-based, effective decision-making training tool. To develop such a tool, it is necessary to understand how experts make prioritisation decisions. This study aimed to model expert decision-making policy for prioritising dietetic referrals in adult acute-care services.

**Methods/Subjects:**

Social judgement theory was used to model expert decision-making policy. Informational cues and cue levels were identified. A set of case scenarios that replicated dietetic referrals in adult acute services were developed using fractional factorial design approach. Experienced dietitians were asked to make prioritisation decisions on case scenarios. A model was derived using multiple regression analysis to elicit the weighting given to cues and cue levels by the experts when making prioritisation decisions.

**Results:**

Six cues and 21 cue levels were identified, and 60 unique case scenarios were created. Fifty experienced dietitians made decisions on these case scenarios. The 'reason for referral' and 'biochemistry picture' were the two most influential cues, and 'weight history' was the least significant. 'Nutritional status', 'presenting complaint' and 'previous food intake' had similar weightings. In all, 95.7% of the variability in the experts’ average judgement (adjusted *R*^2^=0.93) was predicted by the 6 cues.

**Conclusions:**

A model for referral prioritisation in adult acute services described experienced dietitians’ decision-making policy. This can be used to develop training materials that may increase the effectiveness and quality of prioritisation judgements.

## Introduction

Dietitians working in adult acute-care settings often receive large numbers of referrals in batches, making it difficult to see them immediately and making it essential that dietitians have the skills to decide which patients should be seen most urgently. Recent debate in the United Kingdom has focussed on a safe caseload for dietitians^1^ recognising that there is a limit to how many patients any one dietitian can have responsibility for in order for patients to receive safe care. If safe and effective care is to be provided, patients have to be seen according to their clinical need and the urgency for dietetic intervention. Skills in prioritisation are recognised as an 'expert' skill,^2^ which presents difficulties for novices or those new to an area of practice.^3^ Newly qualified dietitians lacking experience or specific training in this skill may struggle to prioritise referrals and feel stressed that they are not meeting patients’ needs. This can have detrimental effects not only on the care provided to patients but also on dietitians’ own health and job satisfaction.

Only two papers are available that look specifically at prioritisation in dietetics. One describes a survey carried out in Australia to establish what systems or tools, if any, were in use and whether they were tested for validity and reliability.^4^ Only 10 hospitals responded and none used evidence-based tools. The other study described the development of a prioritisation tool,^5^ but this was for a specific outpatient oncology service and there was no reliability data. The limited evidence available suggests that there is a need throughout the profession for methods to improve prioritisation and that current systems are generally not evidence based nor well tested.

When experts use and combine information to make decisions, they are applying their own professional 'judgement policy'.^6^ Decision makers can be inconsistent in their judgements, often have limited insights as to how they make judgements and disagree over judgements made.^7^ However, research has shown that it is possible to statistically model how decisions are made, identify judgement policies that produce optimal decisions and use these to improve decision-making capacity of novices.^8, 9, 10, 11, 12^ Very little is known about how experienced dietitians use referral information to make prioritisation decisions, but Harries and Gilhooly^13^ have shown that clinical referral prioritisation of experts can be statistically modelled and that this model can be successfully used to develop effective training materials.^10^ Studies that attempt to identify judgement policies by examining how information is weighted in the decision-making process are known as 'policy-capturing' studies and are conducted within the framework of social judgement theory.^14^ The only identified policy-capturing study in the area of dietetics is a study examining the initiation of artificial nutrition support.^15^

Social judgement theory is ideal for use in decision-making studies where the optimal judgement is not known and where there are real-world consequences when errors are made. Errors in a dietitians’ judgement could mean a delayed intervention, which may impair effectiveness of treatment. Hence, this approach is appropriate for studying dietetic referral prioritisation as there are no 'benchmarks' or 'gold standards' to determine whether a correct decision has been made. Social judgement theory is a quantitative approach that uses statistical methods to describe the relationship between the information available and an individual’s judgement and capture and compare decisions made by group of judges.^6^ When dietitians make prioritisation decisions on dietetic referrals, they weigh information or 'cues' as part of the process. These weights can be modelled by asking expert dietitians to make a large number of decisions on a series of cases in which the cue values are known but varied. The weights can then be determined statistically using such approaches as regression analysis or discriminant function analysis. The resulting decision-making model allows for the identification of individual differences in policies, as well as helps determine an overall decision policy.^6^ The expert consensus judgement policy will guide the decision-making process by providing clear guidance on how information needs to be used and combined and can be used to develop training materials. Training to develop prioritisation skills more rapidly and effectively is required for inexperienced staff, as opposed to a triage tool that may be regarded as too prescriptive by more experienced staff.

This study aimed to capture expert knowledge on dietetic referral prioritisation in the adult acute-care setting and use it to develop an expert consensus judgement policy. From this policy, an evidence-based dietetic referral training package could then be developed to upskill newly qualified professionals.

## Materials and methods

A factorial survey was used to investigate experts’ judgement policy. This shows how many pieces of information (cues and cue levels) are used to reach a judgement and the relevant importance of each of the different cue levels.^16^

First, cues and cue levels were identified through an examination of policy documents, professionals’ case experiences and a review of relevant literature.^17, 18, 19^ Six cues were identified and these are shown in Table 1 with their cue levels. To maximise validity of this information, an expert panel of 5 experienced dietitians (>10-year experience in adult acute care) debated and revised the proposed cues to ensure that all necessary cues and cue levels were represented.

Next the hypothetical case scenarios were created using a fractional factorial sampling (using orthoplan module; SPSS, version 16; SPSS Inc., Chicago, IL, USA), which produced a subset of the full combination of all the cues and cue levels. This minimises the number of cases, reducing the burden on the participants but allowing analysis of the effect of different factors on the decision outcome.^20^ As age and gender should not influence prioritisation, all of the cases were described as a 65-year-old patient, with no gender stated. The cues were presented in the scenarios in the order shown in Table 1, and an example of a case scenario is shown here:

You have received a referral for a 65-year-old patient who may require dietetic assessment. The patient’s presenting complaint is Dysphagia and they have screened as High risk of malnutrition. They have been referred for Enteral tube feeding. The referrer reports that the patient is not eating and has Stable weight. The biochemistry results shows normal biochemistry.

Participants were asked to make a judgement about priority for dietetic assessment in response to each case; five options could be chosen and are shown in Table 2.

Sixty scenarios were created after implausible cases were excluded (such as a patient who was eating well, was weight stable but had biochemistry indicating refeeding syndrome) providing 10 case scenarios for every cue (ratio 1:10), which is within the recommended range from 1:5 to 1:10 for reliable estimates of cue weights.^6^ To check cue independence, Lambda coefficients of association (0=no association and 1=perfect association) were calculated giving an average value=0.03, with a non-significant maximum of 0.04, indicating a satisfactory level of independence. Twenty cases were repeated to measure consistency^6^ giving a total of 80 case scenarios. Every third case was selected and added at the end of the main set of cases, so the repeat cases were not obvious to the participant. To avoid order effects, the order of the main set was randomised for each participant.

Brunel University Research Ethics Committee approved the study protocol (14/10/STF/03). Adverts and e-mail invitations were sent through the British Dietetic Association membership communications, reaching all members in the United Kingdom. Those invited to participate were dietetic professionals with at least 6 months of experience, who worked in an acute adult-care environment. In keeping with published studies using this methodology, for example Harries and Gilhooly,^13^ we aimed to recruit at least 40 participants meeting these criteria. Participants who wished to consider taking part in the study were asked to access a dedicated website, where they were given access to the participant information sheet. If they wished to participate, they entered their details and they were sent a password to access the judgement task. Confidentiality was assured to protect both individual participants’ identity and their place of work; consent was implied by participation. Two practice case scenarios were provided on the website before participants were asked to start the full set of 80, to familiarise them with the format. On completion, each participant was sent a modest honorarium on-line gift voucher to thank them for their time. Data collection ran between 15 and 30 May 2014.

### Data analysis

Three approaches to regression analysis were used: (i) full regression model where all of the cues are included to identify the influence of cue levels in predicting the dependent variable, (ii) step-wise approach, where a cue is entered into the model at each step, in order to establish order of importance of each cue for predicting the expert consensus prioritisation decision, and (iii) reduced models, which are obtained by omitting each cue in turn from the full model, in order to establish whether there is a significant change in judgements as a result of the cue excluded, accounting for the other factors present.

Categorical cues were recoded into dummy variables in order to include them in the regression analyses. Each cue with *k* levels is transformed into *k*−1 dichotomous variables; one cue level is chosen as the reference level and is scored '0' for each dummy variable.^21, 22, 23^ The reference levels are indicated by 'superscripted a' in Table 1 and are a point of comparison for the other levels of that cue. When entering or removing a cue to or from the model, the whole block of dummy variables is entered or removed.

*P*-values were used as a measure of whether regression coefficients are significantly different from zero. Normalised squared semipartial correlations were calculated for each cue level as a measure of their relative importance in predicting the average prioritisation judgements for the case scenarios.^6^ The Bonferroni correction was applied to determine an adjusted significance level to account for the fact that multiple *t*-test comparisons were needed. This analysis was carried out for categorical cues that were identified to have significant influence from the incremental F-test for the reduced models.

The level of agreement between prioritisation decisions made by the dietitians was examined using intraclass correlation coefficient (ICC(2,1)). The level of consistency of each participant (intrarater reliability) was examined using intraclass correlation coefficient (ICC(1,1)).^24^

## Results

Fifty dietitians participated in the study, with a mean age of 32 years (s.d. 8.4), of which 94% (*n*=47) were female and 90% (*n*=45) had ⩾2 years experience in adult acute-care settings. Of all the participants, 58% (*n*=29) were of white ethnic background, 92% (*n*=46) lived in England and 96% (*n*=48) were trained in the United Kingdom, whereas 4% (*n*=2) were trained in Australia.

Across the 50 participants, agreement was very high for prioritisation decisions (ICC(2,1)=0.98 (95% confidence interval (CI) 0.97–0.99)). Consistency was also good with intrarater reliability ICC (1,1) being found to be 0.8 (95% CI 0.74–0.82).

**Figure 1:**
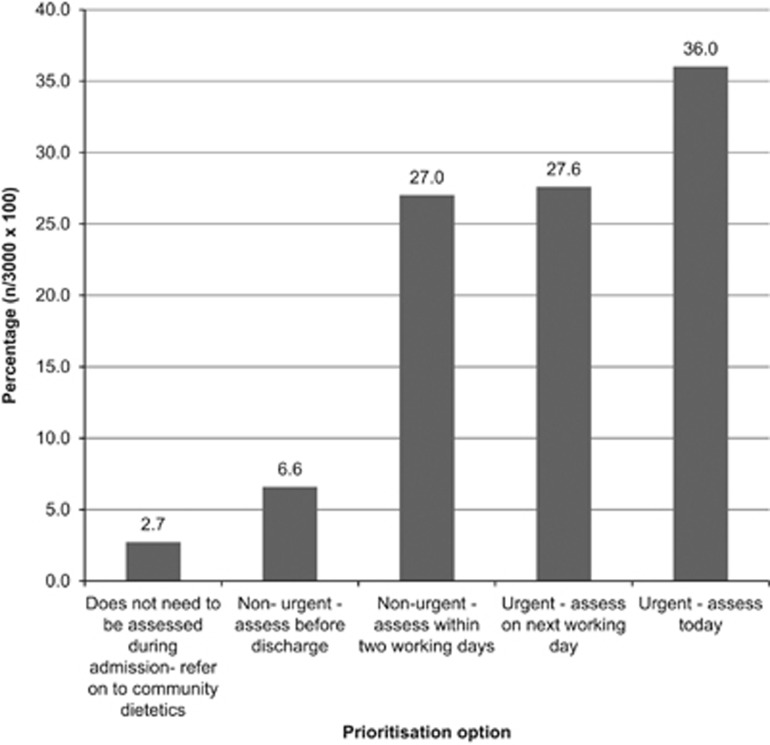
The percentage that each possible prioritisation option was used by the group of 50 experienced dietitians when judging the 60 case scenarios.

Figure 1 shows the frequencies with which each prioritisation option was chosen. Most of the case scenarios were judged as requiring attention within 1–2 days (91% of all judgements), which demonstrates that the cases were representative of a caseload in adult acute services.

Table 3 shows the full regression model (*R*=0.978; F(21, 38)=40.46, *P*<0.001), which accounts for 95.7% of the variance in the experts’ average judgement (adjusted *R*^2^=0.934).

**Figure 2:**
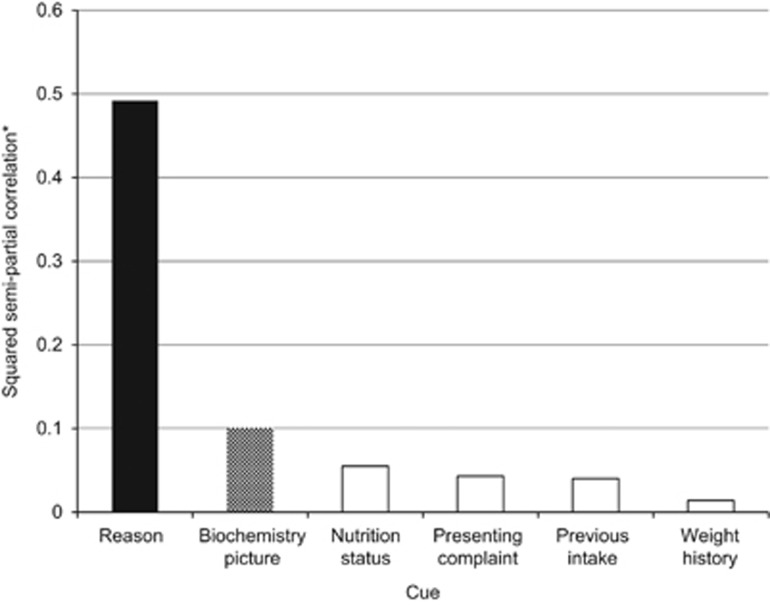
The influence of referral information (cues) on the prioritisation of 60 case scenarios from adult acute-dietetic service referrals. *Illustrates the amount of variability explained by each cue obtained from a reduced model. These values are the additional variability that can be explained by adding each cue into the model with the other five cues already present.

Thirteen of the cue levels had a statistically significant influence on the experts’ average judgements (shaded) and the normalised squared semipartial correlations indicate the relative importance (amount of variability explained by this cue level in the presence of all other cues and cue levels). Referrals with 'parenteral' or 'enteral tube feeding' as the reason were the most important, followed by 'refeeding syndrome' and 'high risk of malnutrition'. The squared semipartial correlations for each cue overall from the reduced models are shown in Figure 2, indicating that 'reason for referral' is the most important cue followed by 'biochemistry picture'. These findings are confirmed in the step-wise regression, with 'reason for referral' alone explaining 61% of the variation and the inclusion of 'biochemistry picture' and 'nutrition status' explaining 86.1% of the variability in the dependent variable (Table 4). The remaining three cues continued to improve the model, but their contributions were much smaller. The reduced models also confirm this pattern of the importance of each cue (data not shown).

In order to further examine the significance between the different levels of the two most important cues: 'reason for referral' and 'biochemistry picture', we made each level as the reference level and inspected the results of *t*-tests for the regression coefficients. The results showed that referrals with either 'enteral tube feeding' or 'parenteral nutrition' as the reason for referral were prioritised significantly higher than the other three cue levels (specialist diet, dietary education or oral nutrition support). The way in which these referrals were prioritised also support the conclusion that these two cues are particularly influential: 64% prioritised as 'Urgent—assess today' and 27% as 'Urgent—assess on next working day'. The referrals with 'dietary education' were not prioritised as significantly more urgent than cases with 'specialist diet' or 'oral nutrition support'. For 'biochemistry picture' this analysis showed that referrals with 'refeeding syndrome' were prioritised as significantly more urgent than cases with 'liver impairment' or 'abnormal K^+^'. There was no significant difference between how 'abnormal K^+^' and 'liver impairment' were prioritised.

## Discussion

A model of expert decision-making policy for prioritising dietetic referrals in adult acute care has been developed, which explains 95.7% of the variability in prioritisation judgements in a group of 50 experienced dietitians. The dietitians involved in this study exhibited a strong consensus in their decisions (ICC(2,1)=0.98) showing that the judgements were reliable. All six cues that were used in the study were found to contribute to the final model but with varying degrees of importance. The 'reason for referral' and 'biochemical picture' were the two most influential cues, explaining >75% of the variability in the average judgements for the scenarios in the step-wise modelling (Table 4). The findings suggest that the most important information to consider when making referral decisions is whether artificial nutrition support is required; this indicates urgency as the patient is entirely reliant on this intervention for nutritional support. The next information to check is the biochemistry; any evidence of a risk of refeeding syndrome should mean these patients are given high priority, due to the detrimental effects associated with the altered biochemistry. The 'nutrition status', 'presenting complaint' and 'previous food intake' had similar importance to each other; any deficit in 'nutritional status', 'poor food intake' or certain presenting complaints ('gastrointestinal cancer' or 'dysphagia') meant a slightly higher priority than if they are not present. The 'weight history' cue was found to be the least important cue in making prioritisation decisions, but the cue-level 'weight loss' slightly increased the priority.

The two papers previously cited in the introduction, which look specifically at prioritisation in dietetics, demonstrate the lack of information in this area. The survey examining prioritisation in Australian hospitals^4^ found that all 10 respondents had a system in place for new inpatient referrals, and these were based on the clinical condition or diagnosis. But this is in contrast to our findings; we found that the experts put most weight on the reason for referral. The tools identified in this survey had been developed through consultation and consensus, with reference to acuity rankings produced by Escott-Stump,^18^ but none had been formally tested for validity and reliability,^4^ and clearly the sample was extremely limited. Nevertheless, there was a belief that the tools brought benefits in terms of helping staff manage workloads, standardising practice among staff, identifying inappropriate referrals, supporting junior staff and ensuring that the high-priority patients would be seen the soonest. The second study, which developed and evaluated a prioritisation tool in oncology outpatients, is of limited use in the adult acute sector. The tool was again based not only on clinical condition rather than reason for referral as we found but also included factors relating specifically to outpatients, such as when the next visit was and how far away the patient lived.^5^ Validity was established by prioritising before and after a full dietetic assessment, but this was not blinded. Results suggested that the tool was able to help dietitians identify the patients with the highest priority, but there were no data on reliability.

The other available evidence on prioritisation comes from other allied health professions; one systematic review shows that all studies in this field are from either physiotherapy or occupational therapy.^25^ The review focusses on systems or tools to help make prioritisation or triage decisions, rather than training professionals to make better decisions. It shows that the research is limited in both quality and quantity, the systems evaluated have only poor to fair reliability and validity is in question, with no tool or system being recommended for use.^25^

Our approach is to understand how the experts make their judgements and use this policy to develop training materials, rather than attempt to develop a decision-making aid, tool or system. Porter and Jamieson^4^ quoted one respondent to their survey as saying 'The senior dietitians tend to use the triage system intuitively without referring to the actual document [triage tool in place in this hospital] whereas the junior staff rely on it quite heavily', suggesting that this is a skill developed with experience. Training to develop these skills more rapidly and effectively is required, rather than a tool that may be regarded as too prescriptive by senior staff. Having developed this judgement policy for experienced dietitians in referral prioritisation, our next step is to test this information’s value in training novices through a randomised control trial.

This is a unique study that is the first to examine expert judgement policy for prioritisation of dietetic referrals. It uses social judgement theory and robust statistical methods to describe a credible model, including evidence of reliability. The cues we used in the cases were based on those validated by a panel of experienced dietitians, supporting the validity of the study design. Nevertheless, there are some potential limitations inherent in the design of the study.

The sample of experienced dietitians was those with '>6 months of experience working in the adult acute services'. Some may argue that 6 months is not enough time to develop appropriate experience in prioritisation; however, we used this relatively low cutoff to ensure recruitment of a large enough group of experts. Only five of the participating dietitians had <2 years’ experience and the sample group had a very high level of agreement, which does indicate that a consensus has been achieved.

It was important to limit the number of cues to ensure the policy development exercise was not unduly time consuming. However, the cues that dietitians receive in referrals vary between referrals and institutions, and not all types of information in this model may fully represent all the information that could be used. Similarly, each cue was divided into levels, but these were not exhaustive lists. For example, the 'presenting complaint' was limited to the most common diagnoses, and specific diagnoses were grouped under broad headings such as 'gastrointestinal cancer' and 'stroke'. Finally, each cue level needed to make sense in randomly generated case scenarios. For this reason, the cue level 'shows abnormal K^+^' was used rather than 'high K^+'^ or 'low K^+^' in order to be able to create a set of realistic cue combinations.

Our scenarios were gender neutral and standardised for age as neither of these factors should influence prioritisation. Nevertheless, some dietitians may have been considering gender and/or age in their analysis of the scenario and this may have biased their judgements. We are not able to separate this effect but believe, if it did occur, that it is unlikely to significantly influence the results.

The expert consensus judgement policy for prioritising dietetic referrals has been identified and describes how experienced dietitians use and weigh information in order to decide how patients should be prioritised. The policy can now be translated into evidence-based training materials with the aim of improving novices’ ability to prioritise referrals.^10^ Effective prioritisation of dietetic referrals is crucial to ensure best patient care, particularly where resources are constrained. Novice dietitians, who normally do not receive formal training on how to prioritise referrals, may feel insecure and consequently stressed when making such decisions, and this may jeopardise sound judgement. A good training package based on the best practice will enable novice dietitians to learn more quickly the skills to make accurate prioritisation judgements confidently, resulting in potentially improved satisfaction and less stress from this source. Future research is planned to determine whether this judgement policy can successfully be used to train novices to make dietetic referral prioritisations in the same manner as experienced dietitians, in order to ensure translation of this study into practitioners’ skill set and to enable the best practice to be shared among novices.

## Figures and Tables

**Table 1:** Cues and cue levels used in the case scenarios to represent adult acute-dietetic service referrals

*Cue*	*Patient information (cue levels)*
(1) Presenting complaint	Chronic obstructive pulmonary disease^a^ Gastrointestinal cancer Dementia Pneumonia A urinary tract infection Falls A stroke Dysphagia Pressure ulcers
(2) Nutrition status (from screening tool)	High risk of malnutrition^b^ At risk of malnutrition^b^ No current risk of malnutrition^a, b^
(3) Reason for referral	Oral nutrition support (food+/− supplements)^a^ Provision of a specialist diet Enteral tube feeding Parenteral nutrition Dietary education
(4) Previous food intake	Is not eating Has poor food intake Is eating well^a^
(5) Weight history	Lost weight Gained weight Stable weight^a^
(6) Biochemistry picture	Shows abnormal K^+^ Suggests refeeding syndrome Suggests liver impairment Shows normal biochemistry^a^

a Indicates the designated reference category for this cue in the regression analysis.

b Malnutrition is defined as underweight or undernourished.

**Table 2:** Priority options available for each scenario

1	Does not need to be assessed during admission—refer on to community dietetics
2	Non-urgent—assess before discharge
3	Non-urgent—assess within 2 working days
4	Urgent—assess on next working day
5	Urgent—assess today

**Table 3:**
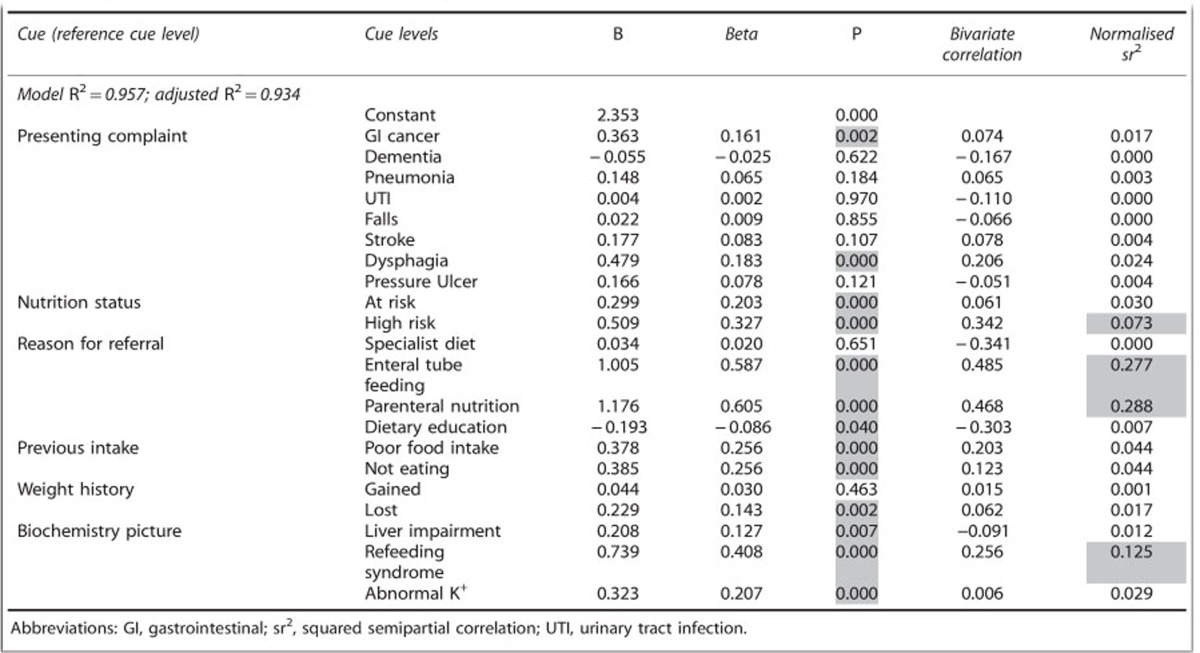
Regression analysis to predict experts’ average referral prioritisation

**Table 4:** Results of the step-wise regression

*Step*	*Cue added*	*Overall model*			*Change statistics*		
		R^2^	*Adjusted* R^2^	*s.e. of estimate*	R^2^ *change*	*F change*	P
1	Reason	0.609	0.581	0.47	0.609	21.4	0.000
2	Biochemistry	0.768	0.737	0.37	0.159	11.9	0.000
3	Nutrition status	0.861	0.836	0.29	0.093	16.8	0.000
4	Presenting complaint	0.912	0.876	0.26	0.051	3.0	0.009
5	Previous intake	0.943	0.916	0.21	0.031	10.98	0.000

NB: Weight history is not included as it was the least significant cue. The fit of the full model, including all cues, is already shown in Table 3.
